# Ultrasound-Guided Bilateral Erector Spinae Plane Block vs. Ultrasound-Guided Bilateral Posterior Quadratus Lumborum Block for Postoperative Analgesia after Caesarean Section: An Observational Closed Mixed Cohort Study

**DOI:** 10.3390/jcm12247720

**Published:** 2023-12-16

**Authors:** Bruno A. Zanfini, Mariangela Di Muro, Matteo Biancone, Stefano Catarci, Alessandra Piersanti, Luciano Frassanito, Mariano Ciancia, Flavia Toni, Maria Teresa Santantonio, Gaetano Draisci

**Affiliations:** Department of Anaesthesiology and Intensive Care Medicine, Fondazione Policlinico Universitario “A. Gemelli” IRCCS, 00168 Rome, Italy

**Keywords:** anaesthesia, obstetrical, caesarean section, nerve block, pain, postoperative, erector spinae plane block, posterior quadratus lumborum block

## Abstract

ESP block (ESPB) and posterior Quadratus Lumborum Block (pQLB) have been proposed as opioid-sparing techniques for the management of pain after abdominal surgery. Between December 2021 and October 2022, we conducted a retrospective comparative study at the delivery suite of Fondazione Policlinico Universitario Agostino Gemelli IRCCS, Rome, Italy, to compare the efficacy of ESPB and pQLB in preventing postoperative pain after an elective caesarean section (CS). The primary outcome was total morphine consumption in the first 24 h. Secondary outcomes were time to first opioid request; Numerical Pain Rating Scale (NPRS) at 0, 2, 6, 12 and 24 h; vital signs; adverse events. Fifty-two women were included. The total cumulative dose of morphine was not significantly different between the two groups of patients (*p* = 0.897). Time to first dose of morphine, NPRS values and haemodynamic parameters were not statistically different between the two groups. NPRS values significantly increased (*p* < 0.001) at the different time intervals considered. The need for rescue doses of morphine was lower in the ESPB group compared to the pQLB group (hazard ratio of 0.51, 95% CI (0.27 to 0.95), *p* = 0.030). No adverse event was reported. ESPB seems to be as effective as pQLB in providing analgesia after CS.

## 1. Introduction

An adequate postoperative analgesia after a caesarean section (CS), one of the most commonly performed surgical procedures worldwide [1], should be a priority and aim at balancing the need for an adequate pain relief while minimizing the risk of analgesia-related adverse events [2]. However, regarding a significant proportion of pregnancies, post-CS pain is undertreated, due to fears of the potential transfer of analgesic drugs to breastfeeding neonates and to underestimation of pain. Up to 11% of patients who undergo elective CS report a moderate to severe postoperative pain [3], which can delay recovery, impair mother–child bonding, complicate breastfeeding [4] and lead to persistent postoperative pain [5]. The incidence of persistent pain is affected by both preoperative patient factors (mainly high analgesic needs and coexisting chronic pain problems) and perioperative factors, related to the surgical and anaesthetic management, with the intensity of pain on the first postoperative day being the most important determinant [6]. Since the latter is the most significant modifiable risk factor and may have lasting effects, an aggressive strategy is warranted to reduce postoperative pain. Currently, intrathecal opioids are considered the cornerstone of postoperative analgesia, although they are associated with nausea, vomiting and pruritus, which may reduce patient satisfaction. Moreover, the risk of opioid misuse and delayed maternal respiratory depression compels the identification of opioid-sparing techniques [7]. As part of multimodal analgesic regimens, as suggested by PROSPECT guidelines [8], fascial plane blocks are gaining popularity. Traditionally, the Transversus Abdominis Plane (TAP) Block has been proposed for analgesia in this context, but the lack of extent on the visceral compartment prevents it from being an optimal alternative to intrathecal morphine (ITM). In fact, the ideal fascial plane block should cover both visceral pain from T10–L1 (the uterus segment) and somatic pain from T11–12 (site of the Pfannenstiel incision) to adequately address pain after CS. The Quadratus Lumborum Block (QLB) and the Erector Spinae Plane Block (ESPB) performed at a low thoracic level have therefore been proposed as promising techniques to manage pain after CS.

Initially developed for thoracic neuropathic pain [9], ESPB has been extensively performed for postoperative analgesia in both open abdominal and laparoscopic surgery [9,10,11,12,13]. From the site of injection (i.e., the plane between the deep erector spinae muscle and its investing anterior sheath wall [9,14,15,16,17]), in ESPB, a local anaesthetic (LA) seems to spread widely craniocaudally and anteriorly, thus resulting in a multi-dermatomal sensory block of the posterior and anterolateral wall of the torso and in visceral pain relief. On the other hand, in posterior Quadratus Lumborum Block (pQLB), LA dissects the plane behind the QL muscle [18], spreading along the middle layer of the thoracolumbar fascia. In this way, it provides somatic and visceral analgesia of the T10–L3 dermatomes, by respectively affecting subcostal nerves and indirectly the thoracic paravertebral space. 

The aim of our study is to assess the efficacy of bilateral thoracic ESPB compared to bilateral posterior QLB (pQLB) for the management of postoperative pain after CS conducted under spinal anaesthesia without ITM.

The study’s primary outcome was the comparison of total doses of morphine postoperatively delivered through a Patient-Controlled Analgesia (PCA) system during the first 24 h from performance of the analgesic block.

Secondary outcomes were the evaluations of the time of the first opioid request, of the pain scores reported by patients, of the haemodynamic parameters and of adverse events.

## 2. Materials and Methods

The study was a single-centre retrospective comparative study conducted between December 2021 and October 2022 at the delivery suite of Fondazione Policlinico Universitario Agostino Gemelli IRCCS, Rome, Italy, in accordance with Good Clinical Practice guidelines and the principles of the Declaration of Helsinki. Ethical approval was provided by the Institutional Independent Ethics Committee of our institution (ID 4412 protocol N.0044540/21). The study was registered on ClinicalTrials.gov (accessed on 15 December 2021) (NCT05348083). All patients provided written informed consent during the pre-anaesthesia visit. Eligible participants were adult women with American Society of Anaesthesiologists (ASA) physical status 2, with normal singleton term pregnancy, scheduled for elective CS under spinal anaesthesia. Exclusion criteria were ASA status ≥ 3, coagulation disorders, emergency surgery, preoperative infection (including infection at the puncture sites for pQLB and ESPB), any contraindication to neuraxial analgesia, history of chronic pain, opioid use, allergy to LA, hypersensitivity to any drug used in the study, inability to comprehend or use verbal pain assessment scales or PCA pump, refusal to participate in the study. Data from the ESPB cohort were compared with the retrospective cohort of patients from a precedent study [19], enrolled between July and December 2020, who received pQLB after elective caesarean delivery and who were included following the same eligibility criteria, perioperative management and data collection. A minimum sample size of 24 patients per group was needed to detect a statistical difference in the primary outcome. 

Upon arrival in the operating room, a peripheral line was placed and patients intravenously received a proton pump inhibitor (e.g., 40 mg omeprazole), 10 mg metoclopramide and antibiotic prophylaxis. All subjects were monitored with pulse oximetry, ECG, non-invasive blood pressure and external tocodynamometry monitors. As per routine hospital practice, spinal anaesthesia was performed in a sitting position using a midline approach into the L3–L4 or L4–L5 interspaces with a 25 G pencil-point spinal needle, administering 9 mg of 0.5% hyperbaric bupivacaine and 5 mcg sufentanil. An adequate extent of the sensory blockade was considered at the bilateral T5 dermatomal level, assessed through loss of pinprick sensation. Upon delivery, unless contraindicated, all patients received 4 mg dexamethasone, 1 g acetaminophen, 30 mg ketorolac or 100 mg ketoprofen intravenously, as per standard practice. At the end of surgery, the fascial blocks were performed by one of two experienced anaesthesiologists as follows:

### 2.1. ESPB Group

ESPB was performed in the lateral decubitus position under ultrasound guidance in a sterile manner, after cleaning the skin with a surgical solution (ChloraPrep, Carefusion, 244 Ltd., Basingstoke, UK). A high-frequency linear US probe (13–6 MHz, Sonosite M-turbo, FUJIFILM Sonosite Europe, Amsterdam, The Netherlands) was placed on a parasagittal plane at the T9 level (sonographically assessed) and slid slightly in the lateral direction until the transverse process was visible. The probe was then placed at the tip of the T9 transverse process, around 3 cm to the side of the midline. Next, the needle (Stimuplex ultra 360, 20 G, 100-mm-long, Braun, Melsungen, Germany) was advanced craniocaudally with an in-plane technique and a 30–45 degree angle. After making contact with the transverse process, the appropriate positioning of the needle tip was assessed alternating aspiration with hydrodissection, injecting 2 mL of normal saline. Lastly, a mixture of 0.375% ropivacaine and 5 mcg/mL epinephrine (20 mL per side) was injected, and the linear spread of LA was confirmed in both a cranial and caudal direction.

### 2.2. pQLB Group

pQLB was performed with the patient in the supine position with a lateral tilt, after cleaning the skin with a surgical solution (ChloraPrep, Carefusion, 244 Ltd., Basingstoke, UK) with a sterile technique, using a Sonosite M-turbo echograph (FUJIFILM Sonosite Europe, Amsterdam, The Netherlands) and a broadband (5–8 MHz) convex probe. The probe was placed in the transverse plane at the level of the anterior superior iliac spine and moved cranially until the three abdominal wall muscles were identified. The external oblique muscle was then followed posterolaterally until its posterior border was found. Next, the probe was tilted down to identify the bright hyperechoic line corresponding to the intermediate layer of the thoracolumbar fascia. The needle (Ultraplex 360, B.Braun, Melsungen, Germany) was inserted in the plane from anterolateral to posteromedial through the muscle layers of the abdominal wall, aimed to the lumbar interfascial triangle (posterolateral aspect of the quadratus lumborum muscle). The optimal needle tip position was assessed through hydrodissection, injecting 2 mL of normal saline. A mixture of 0.375% ropivacaine and 5 mcg/mL epinephrine (20 mL per side) was then administered, ensuring that the spread was posteromedial. 

Patients were discharged from PACU to the hospital ward after a period of 60 min, granted that an Aldrete score of 9 was attained, as per the anaesthesiologist’s order [20]. Throughout the first 24 postoperative hours, patients received 1 g acetaminophen at 8 h intervals intravenously and 30 mg ketorolac or 100 mg ketoprofen bid, according to the obstetric department protocol. All patients also received PCA with morphine at 1 mg/mL (CADD-Solis 2110 Infusion System, Smiths Medical ASD, Inc., Plymouth, USA, 1 mL bolus on demand, no background infusion, 7 min lockout, max of 8 mg/h), as a rescue analgesia strategy. 

### 2.3. Outcomes

The primary outcome was the comparison of the analgesic efficacy between ESPB and pQLB measured as the total number of doses of morphine delivered through the PCA system by the two groups of patients during the first 24 h from performance of the block.

Secondary outcomes included

- Time to first opioid request (interval time between block and first opioid analgesic request);
- The relationship between the analgesic block performed and NPRS (ranging from 0, “no pain”, to 10, “the worst pain imaginable”), at rest and on movement at 0, 2, 6, 12 and 24 h from block performance;
- Differences in haemodynamic parameters;
- Any adverse event, like sedation, nausea and other complications, particularly signs of LA toxicity and the occurrence or persistence of motor weakness at the lower extremities after spinal anaesthesia recovery.

Primary and secondary outcome assessment began immediately after the analgesic block was performed, at standardized times (i.e., t = 0 immediately after block execution, at 2, 6, 12 and 24 h after performance of the block). 

### 2.4. Statistical Analysis

In our precedent study [19], we found a total mean morphine consumption of 5.08 ± 3.12 SD mg in the e-pQLB group in the 24 postoperative hours. Our primary hypothesis was that ESPB performed with the same anaesthetic mixture would result in a clinically relevant reduction of at least 35% of opioid consumption. It was calculated that a sample size of 48 patients, 24 in each group, would provide 80% power to detect this effect using a *t*-test with an α level of 0.05. As the retrospective cohort consisted of 26 patients, a total sample of 52 patients (26 for each group) was finally considered in the present study.

A linear mixed effects analysis was performed to express the relationship between NPRS values at rest and with movement and treatment. The type of analgesic block performed and the time points at which NPRS values were evaluated were entered as the fixed effects (without interaction term) and we added random intercepts for subjects into the model. *p*-Values were obtained through likelihood ratio tests of the full model with the effect in question against the model without the effect. Estimates of predictors used in the model are reported with corresponding 95% confidence intervals (CIs) and *p*-values obtained with the Wald chi-square test. Kaplan–Meier curves and the Cox proportional hazard model were used to analyse the time to the first opioid request. The log-rank test was used to compare the survival times of the two groups. The assumption of proportionality was verified graphically and on the basis of partial Schoenfeld residues while the identification of outliers was based on deviation residues.

Normality distribution of a variable was assessed graphically and with the Shapiro–Wilk test. Continuous data are presented as means with standard deviation or medians with interquartile ranges (25th to 75th IQR). Categorical data are presented as frequencies with percentages. Continuous variables were compared using the two-sample t-test or with the Mann–Whitney U test for independent samples, depending on the distribution of the variable. Categorical variables were assessed with the χ^2^-square test or Fisher’s exact test in case of expected frequencies < 5. The statistical significance level was 0.05%. The data analysis was performed using R (R Foundation for Statistical computing, Vienna, Austria, version 4.1.2).

## 3. Results

Fifty-two patients, twenty-six in each group, were included. No significant differences were found between the two groups in terms of demographic and clinical variables except for a different prevalence of hypothyroidism (19% in ESPB group vs. 0% in pQLB group) and a different duration of surgery (average of 59 min in the ESPB group vs. average of 67 min in the pQLB group), but this difference does not appear to be clinically meaningful. Neonatal outcomes in terms of neonatal weight and Apgar score were comparable between the two groups. Baseline characteristics of the study population and neonatal outcomes are shown in Table 1.

**Figure 1:**
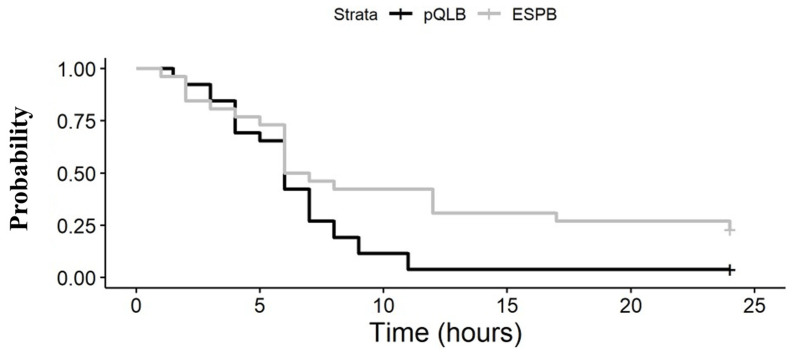
Kaplan–Meier curves for time to first rescue dose of morphine following analgesic block in the pQLB and ESPB group. Censoring refers to the first requested dose of morphine at given time. Abbreviations—ESPB: Erector Spinae Plane Block. pQLB: posterior Quadratus Lumborum Block.

Total cumulative doses of morphine delivered through the PCA system during the first 24 h from performance of the analgesic block were not significantly different between the two groups (4 (IQR 1–12) mg in the ESPB group vs. 4 (IQR 3–6) mg in the pQLB group; *p* = 0.897). The need for rescue doses of morphine was lower in the ESPB group compared to the pQLB group (hazard ratio of 0.51, 95% CI (0.27 to 0.95), *p* = 0.030). In fact, at 24 h, 20 patients (77%) in the ESPB group compared to 25 (96%) in the pQLB group (risk ratio of 0.80 (95% CI: 0.63 to 0.99), *p* = 0.025) required rescue doses of morphine for inadequate analgesia. Among patients requiring rescue morphine, time to the first dose of morphine was not statistically different between the two groups, with a median time of 6 (IQR 3–9) h in the ESPB group compared to 6 (IQR 4–7) hours in the pQLB group (*p* = 0.898) (Figure 1).

**Figure 2:**
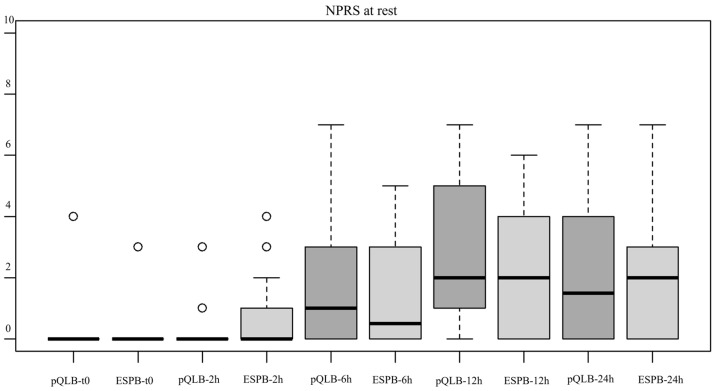
Boxplot showing the distribution of NPRS values at rest for the two groups at different points, t0 = immediately after block execution, 2, 6, 12 and 24 h = after performance of the block. Abbreviations—NPRS: Numeric Pain Rating Scale. ESPB: Erector Spinae Plane Block. pQLB: posterior Quadratus Lumborum Block.

**Figure 3:**
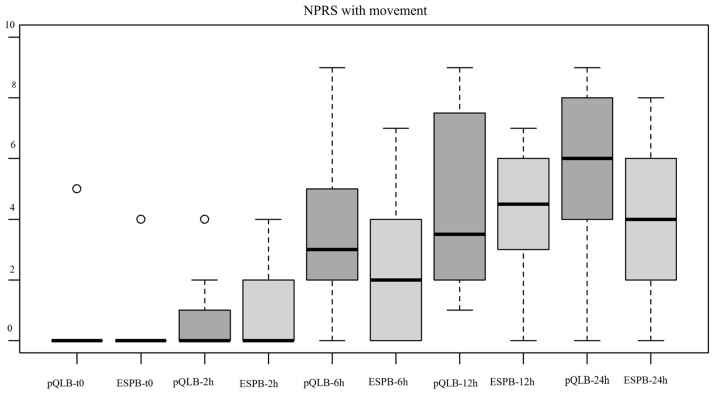
Boxplot showing the distribution of NPRS values with movement between the two groups at different points, t0 = immediately after block execution, 2, 6, 12 and 24 h = after performance of the block. Abbreviations—NPRS: Numeric Pain Rating Scale. ESPB: Erector Spinae Plane Block. pQLB: posterior Quadratus Lumborum Block.

Intensity of pain at rest and with movement was significantly different (*p* < 0.001) at the different time intervals considered, with reported NPRS values increasing as time passed from the execution of the block, but with no statistically significant differences between the two groups both at rest and with movement (χ^2^(1) = 0.68, *p* = 0.407 and χ^2^(1) = 1.79, *p* = 0.180, respectively (Figure 2 and Figure 3)).

No significant differences were reported in haemodynamic parameters and in adverse events between the two groups.

## 4. Discussion

Our results suggest that ESPB has a comparable analgesic effect to pQLB when combined with a multimodal analgesia regimen in parturients undergoing elective CS without ITM. These results are in line with those of a recent trial on 60 patients scheduled for elective CS under spinal anaesthesia and randomised to transmuscular QLB or thoracic ESPB with 20 mL of 0.375% ropivacaine and 4 mg of dexamethasone on each side. The authors found no difference between the two blocks with respect to pain scores, duration of analgesia and use of rescue analgesics [21]. The authors also found a time to first rescue analgesic of 11.90 ± 2.49 h (95% CI: 10.97, 12.83) in group ESPB and 12.56 ± 3.38 h (95% CI: 11.29, 13.82) in group pQLB. These times are considerably longer than those we observed in our cohorts. However, a median time to the first opioid request of 6 h for our patients is consistent with the corresponding rise in NPRS, and adequate analgesia was provided at all times at the expense of a modest dose of morphine (median of 4 mg). One of the major concerns in post-caesarean analgesia is the safety of systemic administered medications for the mother and the breastfeeding infant. However, such a dose is unlikely to cause any negative effects on maternal alertness, and the administration of intravenous morphine is considered safe during lactation, due to the very low passage in milk and the poor bioavailability in the newborn [22]. The reduced need of rescue doses that we observed in the ESPB group may be a chance finding due to the small sample size.

Another RCT [23] comparing 52 patients (26 for each group) who underwent ultrasound-guided QLB type-II or ESPB at the end of CS under spinal anaesthesia similarly exhibited an equal analgesic efficacy, rate of complications and quality of recovery in both groups. The authors found in fact no differences in the 24 h cumulative fentanyl consumption, nor in the trend of NPRS at rest and with movement between the two groups over time. In accordance with our results, the higher pain scores appeared at 12 and 24 h, particularly with movement.

To our best knowledge, the two aforementioned studies [21,23] are the only RCTs comparing the two blocks in this setting. However, their efficacy in preventing postoperative pain after CS has been separately investigated in different studies. Their main mechanism of action seems to be the block of the ventral rami of spinal nerves through the dispersion of LA into adjacent tissues (i.e., the thoracic paravertebral space) [24]: when performed at the T9 level, ESPB seems to assure a dermatomal coverage for T6-L1 levels [12,25,26]; pQLB, on the other hand, is generally believed to extensively cover T10-L3 dermatomes [27,28].

Hu et al. [29] randomised sixty patients scheduled for CS under general anaesthesia to preoperatively receive or not ESPB at the T9 level. ESPB not only improved postoperative analgesia, but significantly reduced the required dose of general anaesthetics and improved intraoperative haemodynamic stability. In another RCT [30] comparing ESPB to Transversus Abdominis Plane Block (TAP) in CS, the authors found that ESPB significantly reduced tramadol consumption and pain at 8 and 12 h, with an increased duration of the block (ESPB, 12 h [10,14] vs. TAP, 8 h [8], *p* < 0.0001). The same results were found by Malawat et al. [31]. As for the comparison with ITM, in a clinical trial [32] on 140 parturients scheduled for CS, randomly allocated to ITM or ESPB at the T9 level, the authors found not significantly higher VAS scores at rest in the ITM group. Recently, in a meta-analysis reviewing 18 RCTs on the role of EPSB in postoperative pain [33], the authors pointed out that ESPB versus no block was associated with lower pain scores and a lower rate of postoperative nausea in abdominal surgery. Another review on the role of ESPB in postoperative pain after CS [34] failed to find a significant reduction in pain scores when ESPB was performed, but showed a lower consumption of tramadol in the ESPB cohort.

With regard to QLB, from its introduction in the field of post-CS anaesthesia in 2015 [18], a large number of trials have been published, comparing QLB to no block, TAP and ITM. In a systematic review and meta-analysis including 12 RCTs [35], the authors confirmed that QLB was effective in reducing opioid consumption and pain scores in patients not receiving ITM, as QLB patients showed significantly lower pain scores at rest and with movement at 12 h. However, QLB seemed not to provide an additional benefit in women receiving ITM. Nonetheless, the comparison between QLB and ITM remains unclear. In a recent RCT on 80 parturients randomised to 0.2 mg ITM, 0.2 mg ITM plus bilateral pQLB or only bilateral pQLB [36] after elective CS, the authors concluded that pQLB in conjunction with ITM yielded a statistically significant longer median pain-free period compared to ITM alone, whereas pQLB alone provided an inferior postoperative pain control compared with ITM. These results are apparently in contrast with a previous RCT on 90 patients scheduled for CS who were randomly allocated to 0.1 mg ITM alone, pQLB alone or the control group [37], in which pQLB was found to provide longer lasting analgesia with lower postoperative opioid requirements. Such a stark difference may be explained by the diverse amount of ITM employed (0.1 mg vs. 0.2 mg).

With regard to the safety profile, we did not observe any sign of LA toxicity or respiratory depression in either group. Apart from a patient in the ESPB group who experienced PONV at 12 h from block performance, there were no cases of postoperative nausea and vomiting (PONV). We did not observe any spinal anaesthesia-related complication in our cohorts. Severe and occasionally life-threatening complications related to neuraxial anaesthesia (subdural hematoma [38], meningitis, permanent neurological damage, chemical arachnoiditis [39]) are so rare in the obstetric cohort that they are difficult to assess, with permanent neurologic complications estimated between 0.3 and 1.2 per 100.000 [40]. Because of its reliability and safety profile, neuraxial anaesthesia is considered the technique of choice in conducting elective CS [41], because it appears safer for the mother (not exposed to general anaesthesia-associated risks, mainly difficult airway management and pulmonary aspiration) and the newborn [42]. General anaesthesia is ultimately reserved when neuraxial anaesthesia is contraindicated or for Category 1 CS.

The absence of any signs of LA systemic toxicity or any serious adverse events is a mainstay when performing blocks in the obstetric population. With the aim of investigating the safety and the efficacy of pQLB in providing analgesia after CS, we conducted an observational study [19] on 52 patients scheduled for CS under spinal anaesthesia without ITM, who underwent pQLB with an anaesthetic mixture of 0.375% ropivacaine (20 mL for each side), with or without epinephrine at 1:200,000. The pQLB cohort in which the block was performed with epinephrine was used for comparison in the present study. In the previous research, we found a statistically significant reduction in the NPRS value at 6 h and in morphine consumption at 24 h when epinephrine was added and, of utmost relevance, a reduction in peak and mean plasmatic concentration of ropivacaine, still in the therapeutic range at each time assessed in both groups. One should remember, though, that the threshold of toxicity for ropivacaine (2.2 mcg/mL) has been established in other settings, and not in the obstetric population, which is more susceptible to LAST syndrome [43] for pharmacokinetic and pharmacodynamic reasons. From a pharmacokinetic point of view, the decreased plasma binding protein concentration and the increased tissue flow reported during pregnancy may lead to a rapid absorption and higher plasma concentrations of free LA. From a pharmacodynamic point of view, pregnancy-induced changes in oestradiol and progesterone levels increase both the sensitivity of neural cells to LA and the irritability of cardiomyocytes, more easily aggravated by the sodium channel blocking action of LA [44]. As a consequence, every caution must be taken to reduce LA plasmatic concentration in the pregnant population. Furthermore, the correct deposition of LA was also indirectly proven by the peak of plasmatic concentration, occurring at 30 min from block performance as expected in fascial plane blocks [45]. In fact, accidental intravenous infusions cause a rather immediate peak plasmatic concentration, with the threshold of toxicity reached the earlier the faster the injection rate and the higher the LA dose [46].

In conclusion, even if they apparently have an equivalent efficacy, we can suggest that ESPB represents a more suitable choice than pQLB for post-caesarean analgesia, as ESPB is technically easier, a major component when performing regional blocks. Abd Ellatif [47] reported a statistically significant shorter performance time for ESPB compared to QLB (mean time of performing block (minute) ± SD: QLB group—9.36 ± 1.03, ESPB group—5.64 ± 0.66, <0.001), possibly due to the absence of risky structures (retroperitoneum, major blood vessels) and the presence of the transverse process as a security fence when performing ESPB. Moreover, even if a proper performance of QLB should not cause motor impairment, the latter is still hypothetically possible through the block of ventral rami of L1-L2 [48], part of the lumbar plexus. This effect is not likely when performing ESPB at the T9 level.

Our study has important limitations. First, it is an observational study with a small sample size, possibly underpowered to detect a difference in terms of safety outcomes. Secondly, we did not measure plasmatic concentration of ropivacaine in the ESPB group. Lastly, we did not analyse long-term outcomes. Future research on the safety profile and on the comparison with 0.1 mg ITM may provide additional insight.

## 5. Conclusions

Ultrasound-guided ESPB performed at the T9 level seems to be as effective as ultrasound-guided pQLB in providing analgesia after elective CS under neuraxial anaesthesia without ITM. ESPB may be easier to perform than pQLB, therefore representing a promising technique to be implemented in postoperative pain management protocols.

## Figures and Tables

**Table 1:** Baseline characteristics of the study population and neonatal outcomes.

Characteristics	ESPB (N = 26)	pQLB (N = 26)	
Age, y	35 (6)	34 (4)	0.667
Height, m	1.63 (6)	1.64 (6)	0.512
Weight, kg	74 (67, 79)	74 (64, 84)	0.777
Body mass index, kg/m^2^	28 (25, 29)	28 (24, 30)	0.843
Medical history			
Gestational diabetes	4 (15)	2 (7)	0.333
Hypothyroidism	5 (19)	0 (0)	0.025
Factor V Leiden mutation	0 (0)	2 (7)	0.245
ASA status 2	26 (100)	26 (100)	>0.999
Gestational weeks	38 (38, 39)	39 (38, 39)	0.588
Primipara	20 (77)	18 (69)	0.531
Neonatal outcomes			
Neonatal weight, g	3175 (374)	3419 (346)	0.053
Apgar score at 1 min	10 (9, 10)	9 (9, 10)	0.511
Apgar score at 5 min	10 (9, 10)	10 (9, 10)	0.954
Surgery			
Pfannenstiel incision	25 (96)	22 (84)	0.158
Joel Cohen incision	0 (0)	1 (4)	>0.999
Length of surgery (minutes)	59 (46, 72)	67 (58, 80)	0.025

Data are presented as N (%), mean (SD) or median (25th to 75th IQR). Abbreviations—ESPB: Erector Spinae Plane Block. pQLB: posterior Quadratus Lumborum Block. ASA: American Society of Anaesthesiologists.

## Data Availability

Data are unavailable due to privacy or ethical restrictions.
